# Development of AI-Based Laryngeal Cancer Diagnostic Platform Using Laryngoscope Images

**DOI:** 10.3390/diagnostics16020227

**Published:** 2026-01-11

**Authors:** Hye-Bin Jang, Seung Bae Park, Sang Jun Lee, Gyung Sueng Yang, A Ram Hong, Dong Hoon Lee

**Affiliations:** 1 Departments of Otolaryngology-Head and Neck Surgery, Chonnam National University Medical School & Hwasun Hospital, Hwasun 58128, Republic of Korea; hyebin117@hanmail.net; 2 Ssang Yong Software Co., Ltd., Gwangju 62070, Republic of Korea; sbmaum@daum.net (S.B.P.); koth940721@gmail.com (S.J.L.); 3 Biomedical Research Institute, Chonnam National University Hospital, Gwangju 61469, Republic of Korea; turbok2@gmail.com; 4 Departments of Internal Medicine, Chonnam National University Medical School & Hwasun Hospital, Hwasun 58128, Republic of Korea; wanilove23@nate.com

**Keywords:** laryngeal cancer, laryngoscopy, deep learning, semantic segmentation, FCN–ResNet101, artificial intelligence

## Abstract

**Objective**: To develop and evaluate artificial intelligence (AI)-based models for detecting laryngeal cancer using laryngoscope images. **Methods**: Two deep learning models were designed. The first identified and selected vocal cord images from laryngoscope datasets; the second localized laryngeal cancer within the selected images. Both employed FCN–ResNet101. Datasets were annotated by otolaryngologists, preprocessed (cropping, normalization), and augmented (horizontal/vertical flip, grid distortion, color jitter). Performance was assessed using Intersection over Union (IoU), Dice score, accuracy, precision, recall, F1 score, and per-image inference time. **Results**: The vocal cord selection model achieved a mean IoU of 0.6534 and mean Dice score of 0.7692, with image-level accuracy of 0.9972. The laryngeal cancer model achieved a mean IoU of 0.6469 and mean Dice score of 0.7515, with accuracy of 0.9860. Real-time inference was observed (0.0244–0.0284 s/image). **Conclusions**: By integrating a vocal cord selection model with a lesion detection model, the proposed platform enables accurate and fast detection of laryngeal cancer from laryngoscope images under the current experimental setting.

## 1. Introduction

Laryngeal cancer is one of the most prevalent malignancies of the head and neck, and its global incidence continues to increase [1,2,3,4,5,6,7,8,9,10,11,12]. Despite advances in both surgical and non-surgical treatment modalities, the prognosis for advanced-stage disease remains unsatisfactory, with the 5-year overall survival rate stagnating at approximately 60% worldwide [1,2,3,4,5,6,7,8,9,10,11,12]. Early detection is therefore essential to improving patient outcomes. Laryngoscopy remains the primary diagnostic tool for laryngeal cancer; however, early-stage lesions are often subtle, and diagnostic accuracy is highly dependent on the clinician’s expertise, which may result in missed or incorrect diagnoses [1,2,3,4,5,6,7,8,11,12].

Artificial intelligence (AI) has demonstrated considerable promise in automating image interpretation across multiple cancer types, including lung, breast, colorectal, and skin cancers [1,2,6,7,9]. In head and neck oncology, AI applications are still emerging, and only a limited number of studies have focused on the detection of laryngeal lesions [1,2,4,8,10]. While these early investigations have confirmed the feasibility of AI-based approaches, they remain constrained by limitations such as small sample sizes, lack of external validation, and insufficient integration into clinical workflows.

In the present study, we developed and validated a novel AI-based diagnostic platform for laryngeal cancer using laryngoscope images. Our goal was to establish a system capable of precise lesion segmentation and classification, thereby supporting clinicians in the early detection of cancer and potentially improving patient prognosis.

## 2. Materials and Methods

### 2.1. Data Sources and Ethics

We retrospectively collected laryngoscope images from patients diagnosed with laryngeal lesions at Chonnam National University Hwasun Hospital between January 2010 and December 2024. All images were retrieved from the hospital’s electronic medical record (EMR) system following approval by the Institutional Review Board (IRB; CNUHH-2024-278, approved on 14 January 2025; and CNUHH-D2025-002, approved on 24 February 2025). Given the retrospective nature of this study and the complete anonymization of data, the requirement for individual informed consent was waived in accordance with institutional guidelines and the Declaration of Helsinki.

Operational definition of vocal-cord visibility: “Vocal cords visible” was assigned when the true vocal folds were identifiable in the frame (i.e., a glottic view was present). “Vocal cords not visible” was assigned when the true vocal folds were not identifiable because the frame predominantly showed non-glottic anatomy (e.g., oral cavity/oropharynx, nasopharynx, supraglottic larynx, hypopharynx, or adjacent structures). Borderline/ambiguous views (partial glottic exposure, secretions, glare, motion blur, or suboptimal scope angle) were conservatively labeled as “not visible.”

All image handling, annotation, and model training pipelines were logged and version-controlled, thereby allowing consistent validation of our AI platform.

Acquisition metadata completeness. For all examinations (2010–2024), the acquisition device and recording software metadata (manufacturer/model and software name/version) were available from institutional records and were extracted for reproducibility (Table 1).

### 2.2. Acquisition Devices and Software

All examinations were performed using flexible laryngoscopy systems. For reproducibility, we documented the exact acquisition and recording configurations used during the study period, including the manufacturer and model of the endoscopy system and the name/version of the recording/capture software (Table 1).

The acquisition system was changed once during the study period: configuration A was used in 2010–2016, and configuration B was used in 2016–2024 (Table 1).

Configuration A: video processor/light source: [PENTAX, CP-1000]; flexible endoscope platform: [PENTAX, VNL9-CP]; video capture/recording unit: [K-NEWTEC, HDR-ONE, V1.0.0.1].

Configuration B: video processor/light source: [PENTAX, EPK-3000]; flexible endoscope platform: [PENTAX, VNL11-J10]; video capture/recording unit: [K-NEWTEC, HDR-ONE, V2.5.1].

All images underwent the same preprocessing pipeline (cropping, resizing, normalization; and CLAHE for lesion detection) as described in Section 2.4 to reduce non-clinical variability.

### 2.3. Dataset for Vocal Cord Selection

**Figure 1:**
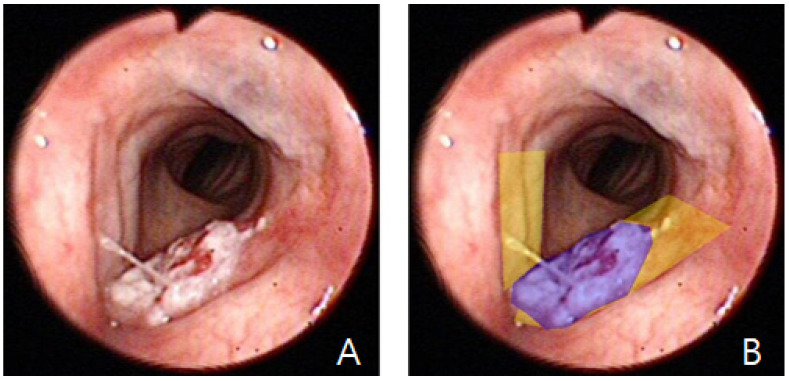
Representative laryngoscope images. (**A**) Original image showing laryngeal cancer lesions. (**B**) Segmentation overlays highlighting the vocal cords (yellow) and laryngeal cancer lesions (purple).

A total of 3617 images from 288 unique patients were collected. Among these, 897 images contained visible vocal cords, while 2720 did not. Two experienced otolaryngologists (HB Jang, with 7 years of clinical experience, and DH Lee, with more than 20 years of experience in head and neck oncology) manually delineated the vocal cord regions using mask-based annotation (Figure 1). Two otolaryngologists independently annotated the images; any discrepancies were resolved by consensus. Formal inter-rater agreement metrics (e.g., inter-rater Dice/IoU) were not computed.

The dataset was randomly divided into training (n = 2894; 80.01%) and test (n = 723; 19.99%) subsets while maintaining class balance. Because examination-session identifiers were not preserved in the anonymized export, the split was performed at the image level (not at the patient/exam/video level). Thus, correlated or visually similar images from the same patient/session may have been present across the training and test sets, which could lead to optimistic performance estimates. Specifically, the training set consisted of 725 vocal cord images and 2169 non–vocal cord images, whereas the test set consisted of 172 vocal cord images and 551 non–vocal cord images. Stratified sampling was applied to ensure the proportion of positive/negative samples remained consistent across datasets.

### 2.4. Image Preprocessing

To minimize background artifacts and emphasize the clinically relevant endoscopic field, a saturation-based cropping strategy was employed. Pixels with saturation ≥ 100 were binarized, the largest connected component was extracted, and the minimal bounding rectangle enclosing this component was applied to crop the original image. This method effectively reduced irrelevant margins, glare, and scope edges.

All images were subsequently padded to maintain aspect ratio and resized to 416 × 416 pixels, followed by channel-wise normalization. For laryngeal cancer lesion detection (Section 2.6), we additionally applied Contrast-Limited Adaptive Histogram Equalization (CLAHE) to enhance mucosal surface texture and boundary contrast. However, preprocessing may introduce appearance changes that could affect cross-device generalizability; therefore, external validation and controlled ablation analyses are warranted in future work.

### 2.5. Data Augmentation

To improve generalization, we applied horizontal flip (*p* = 0.5), vertical flip (*p* = 0.5; selection model only), grid distortion (distort limit = 0.5), and color jitter (brightness/contrast/saturation range = 0.5). Hue was not altered.

Horizontal Flip is an image augmentation technique applied to ensure training data diversity and improve model generalization performance. This technique horizontally flips an image, creating a reversed image. Since the left-right orientation of actual laryngoscope images can vary, including horizontally flipped images helps train the model to be less affected by left-right orientation.

Vertical Flip is an image augmentation technique that vertically flips an image to generate new training data.

Grid Distortion is an image augmentation technique that secures data diversity by deforming laryngoscope images, contributing to improved model generalization performance. This technique divides the original image into grids invisible to the human eye and randomly distorts each cell.

Color Jitter is an image augmentation technique that increases the diversity of training data by randomly altering visual elements such as color, brightness, and saturation in an image. In this study, brightness, saturation, and contrast were randomly varied within a range for vocal cord images to simulate various lighting conditions and visual changes that can occur in real clinical settings.

### 2.6. Model Architecture and Training for Vocal Cord Selection

**Figure 2:**
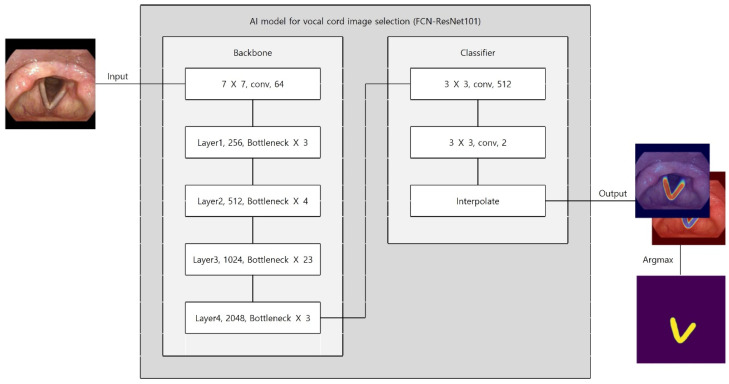
Architecture of the FCN–ResNet101 segmentation model used for vocal cord detection.

We employed a Fully Convolutional Network (FCN) with a ResNet-101 backbone (FCN–ResNet101) for semantic segmentation (Figure 2). The deep residual backbone was chosen for its superior capacity to capture multi-scale contextual features while mitigating vanishing gradient problems. Multi-scale feature maps were derived from intermediate convolutional layers. A classifier assigned probabilities to each pixel (vocal cord vs. background). Bilinear interpolation restored the output mask to the original image resolution. The final mask was generated using an argmax operation on class probabilities.

The model was trained for 700 epochs using the Adam optimizer with an initial learning rate of 10^−4^ for the first 50 epochs and 10^−5^ thereafter. Training utilized a batch size of 4, and optimization was guided by binary cross-entropy loss. To maximize training data usage, we did not create a separate validation set and used a single fixed 8:2 train/test split. The test set was reserved for final evaluation only (i.e., not used for iterative tuning). Therefore, validation curves and validation-based early stopping were not applicable in this study.

### 2.7. Dataset and Training for Laryngeal Cancer Detection

Using the previously trained selection model, we compiled a second dataset comprising 1078 vocal cord images (433 laryngeal cancer; 645 normal). All cancer-positive cases were confirmed by histopathology. “Negative” cases were operationally defined as cases with (1) normal findings on endoscopic examination or (2) benign inflammatory findings (e.g., laryngitis) on endoscopy, with no suspicious lesion identified on follow-up endoscopic examinations documented in the clinical record. Two otolaryngologists (HB Jang and DH Lee) annotated lesion regions (Figure 1), and consensus annotations were used for training.

**Figure 3:**
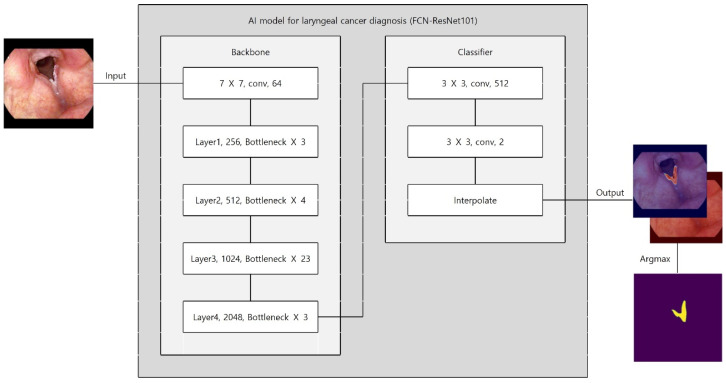
Architecture of the FCN-ResNet101 segmentation model for laryngeal cancer detection.

Preprocessing and augmentation followed the same pipeline as above, except vertical flip was excluded to preserve anatomical orientation critical for lesion detection. An FCN–ResNet101 was trained for 375 epochs using Adam (initial learning rate 10^−4^ for the first 25 epochs, then 10^−5^; batch size 4; binary cross-entropy) (Figure 3). The dataset split maintained class balance (training: 863 images [348 laryngeal cancer; 515 normal]; test: 215 images [85 laryngeal cancer; 130 normal]). As with the selection dataset, splitting was performed at the image level due to the lack of exam/session identifiers in the anonymized export.

This stratified division ensured balanced representation of cancer and non-cancer cases across both subsets.

### 2.8. Evaluation Metrics and Speed

Segmentation performance was assessed using mean Intersection over Union (IoU) and mean Dice coefficient, which are widely accepted in medical image segmentation tasks. For image-level classification, we converted the model output into a binary mask using a per-pixel argmax rule between the two classes (positive vs. background); i.e., a pixel was labeled as positive when the predicted score/logit for the positive class exceeded that for the background class. Under a two-class softmax formulation, this argmax decision is equivalent to thresholding the positive-class probability at 0.5; however, in our implementation, we used the argmax rule directly rather than applying an explicit probability threshold. We then performed connected-component labeling on the predicted positive mask and classified an image as positive if at least one predicted positive component was present (minimum component area, A_min = 1 pixel).

From the resulting confusion matrices, we calculated accuracy, precision, recall (sensitivity), and F1-score. Inference time was measured as a computational benchmark on an NVIDIA RTX 3090 GPU (Hwaseong, Republic of Korea). We did not evaluate end-to-end latency in a deployed clinical workflow or video-pipeline throughput (frames/s).

Threshold-sweep analyses (ROC/PR curves) and sensitivity analyses over alternative thresholds were not performed in this revision and will be addressed in future work.

## 3. Results

### 3.1. Vocal Cord Selection Model

**Figure 4:**
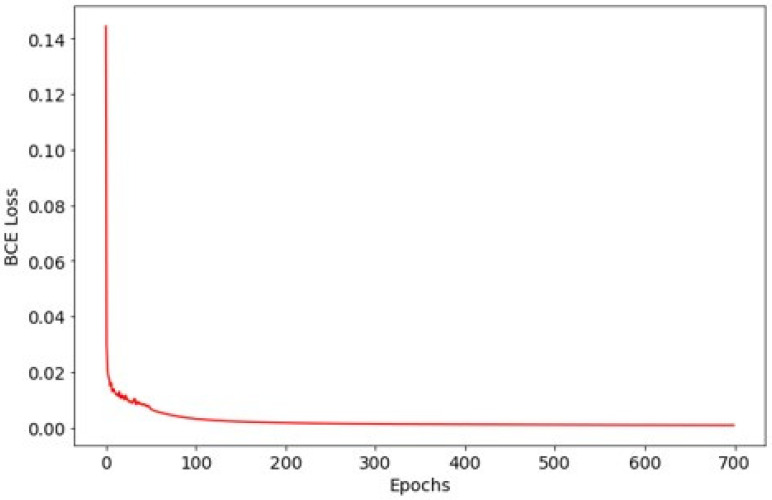
Training loss curve for the vocal cord selection model.

The training loss of the vocal cord selection model decreased steadily from 0.1267 to 0.0008, demonstrating stable convergence without evidence of overfitting (Figure 4). On the independent test set, the model achieved a mean Intersection over Union (IoU) of 0.6534 and a mean Dice similarity coefficient of 0.7692, indicating robust segmentation performance.

When evaluated at the image level, 172 of 174 vocal cord images and 549 of 551 non–vocal cord images were correctly classified. This corresponded to an overall accuracy of 0.9972, precision of 0.9885, recall of 1.0000, and an F1 score of 0.9942. The average inference time was 0.0244 s per image on an NVIDIA RTX 3090 GPU (computational benchmark). End-to-end clinical workflow latency was not evaluated. Such efficiency is critical for integration into routine laryngoscope examinations, where immediate visual feedback may facilitate more accurate lesion localization and improve clinical decision-making.

### 3.2. Laryngeal Cancer Detection Model

**Figure 5:**
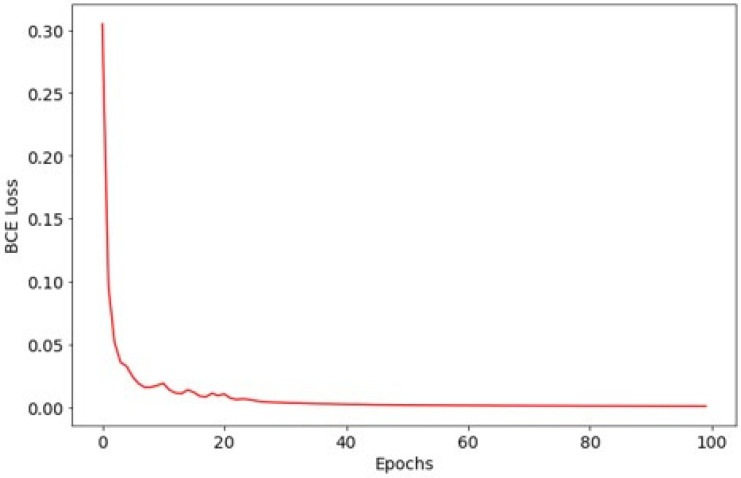
Training loss curve for the laryngeal cancer detection model.

**Figure 6:**
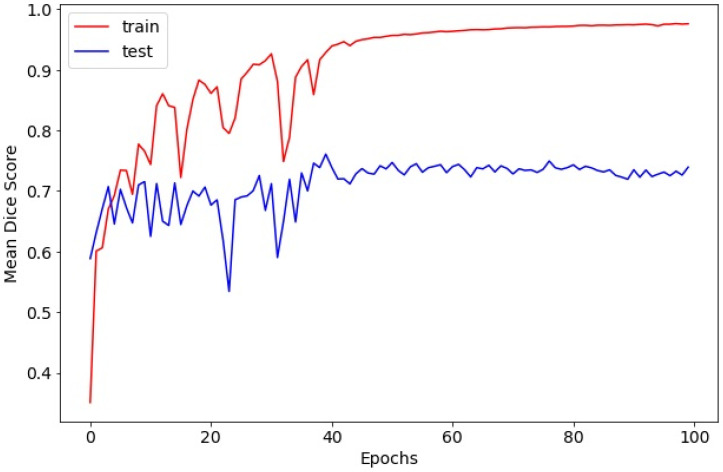
Dice coefficient over training epochs for the laryngeal cancer detection model.

For the laryngeal cancer detection model, training loss decreased from 0.3047 to 0.0000445, again confirming effective convergence (Figure 5). The Dice coefficient over training epochs is shown in Figure 6. On the test dataset, the model achieved a mean IoU of 0.6469 and a mean Dice coefficient of 0.7515 for lesion segmentation.

In image-level classification, the model correctly identified 84 of 85 cancer-positive images and 128 of 130 normal images, resulting in an overall accuracy of 0.9860, precision of 0.9767, recall of 0.9882, and an F1 score of 0.9825. The mean inference time was 0.0284 s per image on an NVIDIA RTX 3090 GPU (computational benchmark).

Importantly, the high recall rate suggests that the model is particularly effective at minimizing false negatives, a critical factor in cancer detection where missed diagnoses may delay treatment initiation. Compared with prior studies that reported lower segmentation accuracy due to smaller datasets and limited external validation, our model demonstrates both strong performance metrics and computational efficiency. This balance of accuracy and speed underscores its potential utility as a clinical decision-support tool in head and neck oncology.

## 4. Discussion

Laryngeal cancer is one of the most common malignancies of the head and neck, accounting for approximately 1–5% of all malignant tumors worldwide [1,2,3,4,5,6,7,8,9,10,11,12]. Early diagnosis is critical, as it not only improves overall survival but also increases the likelihood of preserving laryngeal function [1,2,3,4,5,6,7,8]. Although laryngoscopy remains the standard diagnostic tool for early detection and evaluation of laryngeal lesions, achieving a reliable diagnosis requires a substantial learning curve and remains subject to inter-observer variability [1,2,3,4,5,6,7,8,11,12]

AI, particularly deep learning, has recently emerged as a powerful adjunct in oncologic imaging, with demonstrated efficacy in cancers of the skin, lung, breast, and kidney [1,2,4,7,8,9,10]. Translating this success to the head and neck domain, and specifically to laryngeal cancer, is a natural progression but remains underexplored compared to other cancer types. Our study contributes to this field by developing an AI-based diagnostic platform tailored to laryngoscope imaging, addressing challenges related to early lesion recognition and diagnostic consistency.

The proposed platform integrates two sequential deep learning models—a vocal cord selection model and a laryngeal cancer detection model—both based on the FCN–ResNet101 architecture. This modular design enabled efficient region-of-interest identification followed by targeted lesion segmentation. Both models achieved satisfactory overlap metrics (Dice scores of 0.7692 and 0.7515, respectively) and exceptionally high image-level accuracies (0.9972 and 0.9860, respectively), exceeding or at least matching the performance of prior AI-assisted laryngoscopy studies, which typically reported Dice scores in the range of 0.70–0.75 and classification accuracies around 0.94–0.97 [1,2,4,8,10]. The inference times of 0.0244–0.0284 s per image underscore the system’s capability for near real-time deployment, an essential requirement for clinical integration during live endoscopic examinations.

Several methodological features likely contributed to the observed performance. Preprocessing strategies, including saturation-based cropping and CLAHE enhancement, enhanced lesion boundary visibility, thereby facilitating the detection of subtle, early-stage lesions that are often overlooked in routine practice. The modular two-step architecture also offers flexibility, allowing for the future incorporation of additional tasks, such as classification by histopathological subtype, risk stratification, or prediction of treatment response, without necessitating retraining of the entire pipeline.

From a clinical perspective, this platform holds the potential to mitigate diagnostic variability, assist less experienced clinicians, and expand access to high-quality diagnostic support in resource-limited settings where experienced otolaryngologists may be scarce. Integration into clinical workflows could allow automated lesion highlighting during laryngoscopy, serving as a “second reader” to improve diagnostic confidence. This mirrors the clinical trajectory of AI in radiology, dermatology, and gastroenterology, where AI-assisted endoscopy has already been adopted for polyp detection and skin lesion triage. Recent studies in head and neck oncology have also underscored the importance of AI for improving diagnostic consistency and supporting screening programs, highlighting the growing clinical need for such systems.

Notably, because the split was performed at the image level and the dataset may contain multiple images from the same patient/examination session, correlated or near-duplicate images may have been present across splits. This can inflate image-level performance relative to true patient-/exam-level generalization. Future studies should enforce patient-/exam-level splitting, apply explicit near-duplicate filtering (e.g., perceptual-hash/similarity screening), and perform external multicenter validation.

We reported point estimates of performance metrics; confidence intervals and distributional summaries (e.g., median/IQR) were not provided. Controlled ablation studies for preprocessing/augmentation were also not conducted in this revision. These analyses should be included in future work under patient-/exam-level splits and external validation.

Nevertheless, our work has limitations. Data were collected retrospectively from a single institution, which may limit generalizability. Regarding reproducibility, we now provide the exact manufacturers/models of the endoscopic acquisition systems and the recording software (including versions) used during 2010–2024, and we explicitly document the single equipment change during the study period (Table 1). Nevertheless, performance may still vary across other device ecosystems and institutions; therefore, external multicenter validation remains necessary. Furthermore, the current binary classification framework does not distinguish between benign and malignant lesions, nor does it account for disease stage or histological subtype. Future efforts should expand to multiclass classification and incorporate multimodal imaging, such as narrow-band imaging (NBI) or autofluorescence, to enhance diagnostic specificity. Prospective clinical trials are also warranted to evaluate the system’s impact on diagnostic accuracy, workflow efficiency, and patient outcomes in real-world clinical settings.

## 5. Conclusions

An AI-based platform integrating vocal cord selection and lesion detection enables accurate, efficient, and real-time identification of laryngeal cancer from laryngoscope images. With further validation, the system may assist clinicians in early diagnosis, reduce inter-observer variability, and support treatment planning in diverse clinical settings.

## Figures and Tables

**Table 1:** Endoscopic acquisition devices and recording software used in this study (2010–2024). The acquisition system changed once during the study period; exact device/software identifiers are provided for reproducibility.

	Period	Video Processor/Light Source	Flexible Endoscope Platform	Video Capture/Recording Unit
A	2010–2016	PENTAX, CP-1000	PENTAX, VNL9-CP	K-NEWTEC, HDR-ONE, V1.0.0.1
B	2016–2024	PENTAX, EPK-3000	PENTAX, VNL11-J10	K-NEWTEC, HDR-ONE, V2.5.1

## Data Availability

The data presented in this study are available on request from the corresponding author.
